# Prevalence and implications of the presence of intraocular silicone oil droplets in patients treated with intravitreal injections of anti-VEGF

**DOI:** 10.1186/s12886-022-02536-2

**Published:** 2022-07-26

**Authors:** Sandra Banderas García, Xavier Garrell-Salat, Fernando Trejo-Velasco, David Aragón-Roca, Miguel Ángel Zapata, José García-Arumí

**Affiliations:** 1 Department of Ophthalmology, Vall d’Hebron University Hospital, Passeig de la Vall d’Hebron, 119-129, 08035 Barcelona, Spain; 2 Departament de Cirurgia i Ciències Morfològiques, Universitat Autònoma de Barcelona, Barcelona, Spain

**Keywords:** Intravitreal injections, Silicone droplets, Anti-VEGF, Floaters, Intraocular pressure, Macular diseases

## Abstract

**Background:**

To determine the percentage of patients who have silicone droplets in the vitreous after treatment with different anti-Vascular Endothelial Growth Factor (anti-VEGF) intravitreal injections (IVI) and how symptomatic they are.

**Methods:**

One hundred fifty-two eyes of 140 patients who had at least received an IVI were recruited for this study. Data collection included the number and type of IVI (aflibercept, ranibizumab and bevacizumab) and the follow-up time. A complete ophthalmologic examination was carried out and patients were classified in four groups according to the amount of silicone droplets found in dilated fundoscopy (nonexistent, scarce, moderate and abundant). Measurement of intraocular pressure (IOP) was also carried out. An interview was conducted to report the presence and intensity of the symptomatology.

**Results:**

Silicone oil droplets were reported in 109 eyes (71.7%). A positive correlation was found between the number of IVIs received and the quantity of droplets found, especially when aflibercept was used. Posterior vitreous detachment (PVD) was present in 65.8% of the patients, showing a positive correlation with the number of bubbles. Regarding the symptomatology, 60 eyes (39.5%) had floaters and the disturbance was reported to be 4 out of 10. The group with a moderate amount of silicone droplets had the highest percentage of floaters (60%). No statistical differences in the IOP were found between groups, although the group with abundant droplets had a higher mean IOP.

**Conclusion:**

A high prevalence of silicone droplets in vitreous of patients who undergo IVI treatment was found. It appears to have little impact on symptomatology and rise of IOP.

## Background

The intravitreal injection of drugs that inhibit vascular endothelial growth factor (anti-VEGF) is the first therapeutic option in the treatment of choroidal neovascularization that appears in age-related macular degeneration (AMD), as well as in the treatment of diabetic macular edema and macular edema secondary to retinal vein occlusion. Most patients receive several of these injections with a frequency that can vary between monthly and quarterly, depending on the severity of the macular damage they present, becoming a chronic treatment for a large percentage of these patients.

The prevalence of some forms of AMD and diabetic retinopathy are 1.5% of the general population and 35% of the diabetic population, respectively; and the expected growing trend in the coming years will lead to an immense number of patients exposed to these droplets of silicone oil [1]. Since 2006, the presence of silicone oil droplets has been observed in the vitreous of those patients who received intravitreal injections [2] and the quantity of these is directly proportional to the number of doses received [3, 4]. Macro-sized droplets (larger than 100 μm) are typically visible to naked eye, while micro-sized droplets (up to 60 μm) can only be observed with technologies such as imaging flow cytometry [5]. In our study, we will always refer to macro-sized droplets because of the examination technique employed. Silicone oil is used in the manufacture of disposable syringes, in which the medication is pre-filled, to lubricate the piston and thus prevent it from sticking to the syringe tube.

Although these droplets generally do not generate symptoms, a percentage of patients report transient golden floaters for an average of 1 week after intravitreal injection. On the other hand, silicone oil when used as a plugger in retinal detachment surgeries can pass into the anterior chamber and cause ocular hypertension by blocking the iridocorneal angle. Most commercially available needles also are siliconized in their inner lumen [5].

The objective of our study was to determine the percentage of patients who have silicone droplets in the vitreous after treatment with anti-VEGF intravitreal injections and how symptomatic they are for the patient.

## Methods

### Inclusion and selection of patients

This was a transversal study, which was approved by the clinical research Ethics Committee of our center. The tenets of the Declaration of Helsinki were followed and an informed consent was obtained from the subjects.

We included patients who agreed to participate in the study, were older than 18 years old, had received at least 1 intravitreal injection of any anti-VEGF agent and had a visual acuity better than hand-motion (0.005 in Snellen Scale). We decided to take as sample all patients with a programmed appointment for intravitreal injections of the Ophthalmology Department of the Vall d’Hebron University Hospital (Barcelona, Spain) within 5 weeks between 1st of February and 4th of March 2021. Exclusion criteria were the impossibility of signing the informed consent, eyes with prior vitrectomy, presence of vitreous hemorrhage, synchysis scintillans or asteroid hyalosis, known history or actual active uveitis, poor mydriasis or severe allergic reaction to tropicamide, fluorescein or oxybuprocaine.

### Examinations performed

**Fig. 1:**
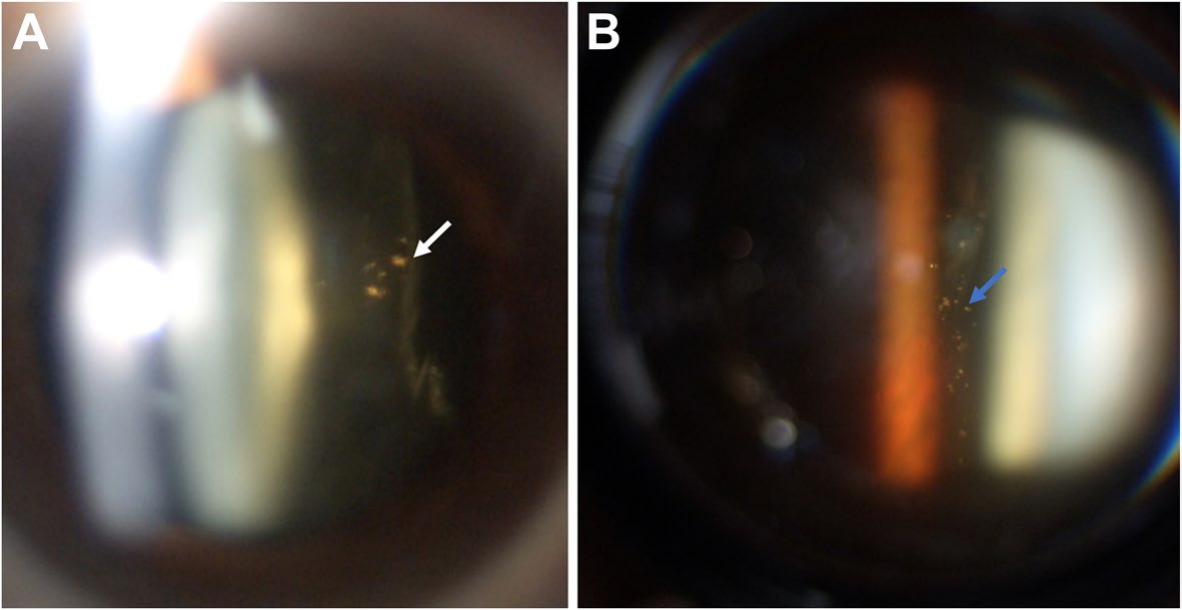
A careful slit-lamp examination with pharmacologic mydriasis reveals the presence of numerous silicone oil droplets (white arrow) in the anterior vitreous (**A**) and in the posterior vitreous (**B**) with blue arrow

The examinations were performed at least 1 month after the last intravitreal injection to the patient. The following variables were recorded from the patient files: age, sex, pathology treated, follow-up time since the first intravitreal injection, and number and type of intravitreal agents received. Consecutively, a careful examination at slit-lamp by an experienced ophthalmologist (DAR, SBG, XGS and FTV) was performed with pharmacologic mydriasis (one drop of tropicamide 0.5%, single dose 10 minutes before the examination) to determine the presence of silicone bubbles in anterior vitreous and with a superfield lens for bubbles in the posterior vitreous (Fig. 1). PVD was also ruled out: visualization of posterior hyaloid membrane with absence of gel posterior to it. Measurement of intraocular pressure (IOP) by means of Goldmann tonometry was carried out. Every patient was then classified into four categories: “Non-existent”, “Scarce” (less than 10 bubbles in vitreous), “Moderate” (between 10 and 30 bubbles) and “Abundant” (more than 30 bubbles). Following the examination, a short interview was conducted asking for symptoms including: vision of floaters, presence of a central scotoma and metamorphopsia. If the answer to any of the last symptoms was positive, a scale from 1 to 10 was used to indicate the intensity of the disturbance that that symptom would cause.

### Characteristics of the agents

Aflibercept (Eylea: Regeneron Pharmaceuticals, Inc., Tarrytown, NY, US) is administered to our hospital in a vial which is split by the pharmacy of our center into 3 sterile disposable 0.3 mL BD Microfine + Demi syringes (BD, Franklin Laked, NJ, US). Each syringe is filled with 0.06 mL of Eylea. These are stored between 5 °C and 8 °C before use. Recently a prefilled glass syringe has been commercialized and used in our center.

Ranibizumab (Lucentis: Genentech, San Francisco, CA, US) consists of a single-use, sealed sterile tray prefilled glass syringe containing 0.165 mL of sterile solution. The top is fabricated from bromobutyl recovered with a white rubber cover stopper and includes a Luer Lock adapter. These are stored between 5 °C and 8 °C before use.

Bevacizumab (Avastin: Genentech, San Francisco, CA, US) is repacked from vials by the compounding pharmacy of our hospital and then filled into 20 1.0 mL sterile disposable Luer Lock syringes (DH material médico, Barcelona, Spain). Each syringe is filled with 0.2 mL of Avastin. These are stored between 5 °C and 8 °C before use.

Dexamethasone implant (Ozurdex: Allergan Pharmaceuticals, Ireland) is a single use biodegradable intravitreal implant with 0.7 mg of dexamethasone located in the stainless-steel needle of a disposable applicator. The product does not require of any special storage conditions.

### Statistical study

Statistical analyses were performed with the SPSS (Armonk, NY, US, v23.0). The normality of the variables was tested with the test of Kolmogorov-Smirnov. The quantitative variables that followed a normal distribution were studied with analysis of variance (ANOVA) and if significant, groups were separately tested using Bonferroni correction. Kruskal-Wallis test was performed to compare quantitative variables not normally distributed and if differences found to be significant, groups were tested separately using Mann-Whitney U test. Qualitative variables were examined using the Χ^2^ test. Spearman correlation was used to explore correlation among variables. *P* < 0.05 was considered statistically significant.

## Results

### Study population

We included a total of 140 patients and 152 eyes. Men accounted for 53.3% of the patients were men and mean age was 73.6 years. The mean follow-up time since the beginning of intravitreal treatment in our center was 34.7 months and the mean intravitreal injections per eye was 10.11. The total amount of intravitreal injections was of 1544 with 876 aflibercept (56.7%), 553 ranibizumab (35.8%), 86 bevacizumab (5.6%) and 29 dexamethasone intravitreal implants (1.9%). More characteristics from our sample are available in Table 1.

**Table 1:** Demographic features of the patients’ data

*N* = 152	Descriptive
Eye (right, %)	79 (51.9%)
Sex (male, %)	82 (53.3%)
Age (mean, SD)	73.6 (SD 10.72)
Pathology (number,%)	
AMD	85 (55.9%)
RVO	26 (17.1%)
DME	24 (15.8%)
Myopia	4 (2.6%)
Others	13 (8.6%)
Snellen VA (mean, SD)	0.5 (SD 1.29)
Follow-up time in months (mean, SD)	34.68 (SD 30.74)
IVI number (mean, SD)	1544 (10.1, SD 8.45)
Aflibercept number (mean, SD)	876 (5.8, SD 5.94)
Ranibizumab number (mean, SD)	553 (3.6, SD 5.77)
Bevacizumab number (mean, SD)	86 (0.6, SD 1.53)
Dexamethasone implant number (mean, SD)	29 (0.2, SD 0.99)
Presence of PVD (n, %)	100 (65.8%)
Symptoms	
Scotoma (n, %)	30 (19.7%)
Metamorphopsia (n, %)	52 (34.2%)
Floaters (n, %)	60 (39.5%)
IOP (mean, SD)	16.07 (SD 8.81)

*SD* Standard deviation, *AMD* Age-related macular degeneration, *DME* Diabetic macular edema, *IOP* Intraocular pressure, *IVI* Intravitreal injections, *PVD* Posterior vitreous detachment, *RVO* Retinal vein occlusion, *VA* Visual acuity

A total of 109 eyes (71.7%) presented silicone oil droplets in the vitreous. Of these, 40 eyes (26.3%) accounted for the “Scarce” bubbles group, 35 eyes (23.0%) for the “Moderate” group and 34 eyes (224%) for the “Abundant” group. We examined differences between groups in terms of age, sex, visual acuity, pathology or follow-up time (Table 2), with no statistically significant differences. Differences were found in terms of the presence of PVD among all groups (global *p* = 0.003). When examining this difference, statistically significant proportions were found between the “Scarce” and “Abundant” groups (47.5% vs. 88.3% with *p* < 0.05).

**Table 2:** Main features in the four groups. Statistical analysis in the last column

	Non-Existent *N* = 43	Scarce *N* = 40	Moderate *N* = 35	Abundant *N* = 34	Significance among groups
Age (mean, SD)	71.84 (SD 1.89)	74.60 (SD 1.65)	70.77 (SD 1.59)	77.71 (SD 1.51)	*p* = 0.13^a^
Male sex (number, %)	23 (53.5%)	19 (47.5%)	24 (68.6%)	15 (44.1%)	*p* = 0.174^b^
Snellen VA (n, SD)	0.32 (SD 0.03)	0.44 (SD 0.4)	0.92 (0.44)	0.34 (SD 0.04)	*p* = 0.084^a^
Pathology (n, %)					*p* = 0.243^b^
AMD	19 (44.2%)	27 (67.5%)	19 (54.3%)	20 (58.8%)	
RVO	5 (11.6%)	6 (15%)	7 (20%)	8 (23.5%)	
DME	9 (20.9%)	5 (12.5%)	6 (17.1%)	4 (11.3%)	
Myopia	2 (4.6%)	1 (2.5%)	0 (0%)	1 (2.8%)	
Other	8 (18.6%)	1 (2.5%)	3 (8.6%)	1 (2.8%)	
PVD Present (n, %)	27 (62.8%)	19 (47.5%)	24 (68.6%)	30 (88.2%)	***p*** **= 0.003**^b^
IOP (mean, SD)	15.21 (SD 0.515)	16.05 (SD 0.765)	14.63 (SD 0.499)	18.68 (SD 2.94)	*p* = 0.259a
Follow-up time in months (mean, SD)	27.79 (SD 4.97)	36.05 (4.61)	39.03 (5.4)	37.32 (SD 4.83)	*p* = 0.066^a^

*SD* Standard deviation, *VA* Visual acuity, *RVO* Retinal vein occlusion, *AMD* Age-related macular degeneration, *DME* Diabetic macular edema, *PVD* Posterior vitreous detachment, *IOP* Intraocular pressure

Statistically significant *p* < 0.05 in bold

^a^Kruskal-Wallis

^b^Χ^2^ test

### Intravitreal agent

The average number of intravitreal injections that the patients with no silicone droplets received was 6.26, while the patients with droplets had received a mean number of 11.70 intravitreal injections (Table 1). Statistically significant differences were found regarding the mean number of intravitreal injections when comparing groups (*p* < 0.001). All 3 groups with silicone oil bubbles had received a significantly bigger mean number of injections when compared with the “Non-existent” droplets group (“Non-existent” vs. “Scarce” *p* = 0.001; “Non-existent” vs. “Moderate” *p* < 0.001; “Non-Existent” vs. “Abundant” p < 0.001). Moreover, a positive moderate correlation was found between the average number of injections and the group regarding the number of bubbles (Spearman correlation rs = 0.330; *p* < 0.001).

Regarding the intravitreal agents that were used, we analyzed the average number of aflibercept, ranibizumab, bevacizumab and dexamethasone injections that each group had received (Table 3). Statistically significant differences were found for aflibercept (*p* < 0.001) and ranibizumab (*p* = 0.006). Examining the correlation between the average number of intravitreal injections of these two drugs and the group regarding the number of droplets by means of Spearman correlation factor, we detected a negative weak correlation for ranibizumab (rs = − 0.228; *p* = 0.005) and a moderate positive one for aflibercept (rs =0.574; *p* < 0.001).

**Table 3:** Mean number of anti-VEGF injections used in each group

	Non-existent	Scarce	Moderate	Abundant	Significance among groups
Total number of intravitreal injections (n, SD)	6.26 (SD 1.06)	10.05 (SD 1.06)	13.09 (SD 1.77)	11.97 (SD 1.40)	***p*** **< 0.001**^a^
Aflibercept intravitreal injection (n, SD)	1.51 (SD 0.33)	5.82 (SD 0.73)	7.45 (SD 1.02)	9.32 (SD 1.23)	***P*** **< 0.001**^a^
Ranibizumab intravitreal injection (n, SD)	4.40 (SD 0.97)	3.78 (SD 0.790)	4.97 (SD 1.22)	1.15 (SD 0.49)	***p*** **= 0.006**^a^
Bevacizumab intravitreal injection (n, SD)	0.30 (SD 0.113)	0.30 (SD 0.169)	0.51 (SD 0.176)	1.26 (SD 0.451)	*p* = 0.069^a^
Dexamethasone intravitreal implant (n, SD)	0.14 (SD 0.140)	0.23 (SD 0.166)	0.14 (SD 0.083)	0.26 (SD 0.236)	*p* = 0.702^a^

Statistically significant *p* < 0.05 in bold

^a^Kruskal-Wallis and Mann-Whitney U test

### Symptomatology

In our interview, a total of 30 eyes (19.7%) had a central scotoma, 52 eyes (34.2%) had metamorphopsia and 60 eyes (39.5%) had floaters (Table 1). We compared the presence of these symptoms in each group regarding the number of bubbles that were found. No differences were found among groups when analyzing the presence of central scotoma or metamorphopsia (Table 4). However, the presence of floaters statistically differed among groups (global *p* = 0.026). Difference was only observed between “Scarce” vs. “Moderate” groups (27.5% vs. 60% with *p* < 0.05).

**Table 4:** Symptomatology reported in each group

	Non-Existent *N* = 43	Scarce *N* = 40	Moderate *N* = 35	Abundant *N* = 34	Significance among groups
Central scotoma Yes (n, %) *N* = 30	12 (27.9%)	7 (17.5%)	6 (17.1%)	5 (14.7%)	*p* = 0.452^b^
Scotoma discomfort (mean, SD)	6.92 (SD 0.88)	5.43 (SD 0.97)	5 (SD 1.36)	6.20 (SD 1.42)	*p* = 0.492^a^
Metamorphopsia Yes (n, %) *N* = 52	17 (27.9%)	13 (32.5%)	12 (34.3%)	10 (29.4%)	*p* = 0.815^b^
Metamorphopsia discomfort (mean, SD)	5.18 (SD 0.64)	5.69 (SD 0.72)	5.08 (SD 0.71)	5.90 (SD 0.87)	*p* = 0.890^a^
Floaters Yes (n, %) *N* = 60	17 (27.9%)	11 (27.5%)	21 (60%)	11 (32.3%)	***p*** **= 0.026**^b^
Floaters discomfort (mean, SD)	3.29 (SD 0.56)	3.91 (SD 0.82)	3.33 (SD 0.45)	3.64 (SD 0.83)	*p* = 0.957^a^

Statistically significant *p* < 0.05 in bold

^a^Kruskall-Wallis and Mann-Whitney U test

^b^X^2^ test

We also examined the possible relationship between the number of intravitreal injections received and the presence of floaters. Although no statistically significant difference among groups was found, the patients with floaters had received a higher number of intravitreal anti-VEGF (Table 5). Similarly, no statistical differences were found when analyzing the presence of floaters in relation to the type of drug used, but patients with floaters had received a higher amount of anti-VEGF intravitreal agents.

**Table 5:** Presence of floaters regarding the intravitreal agent used

	Floaters No *N* = 92	Floaters Yes *N* = 60	Significance
Total number of intravitreal injections (mean, SD)	8.73 (SD 0.71)	12.22 (SD 1.31)	*p* = 0.068^a^
Aflibercept intravitreal injection number (mean, SD)	5.14 (SD 0.49)	6.71 (SD 0.94)	*p* = 0.635^a^
Ranibizumab intravitreal injection number (mean, SD)	2.87 (SD 0.49)	4.82 (SD 0.89)	*p* = 0.131^a^
Bevacizumab intravitreal injection number (mean, SD)	0.54 (SD 0.17)	0.60 (SD 0.16)	*p* = 0.154^a^
Dexamethasone intravitreal injection number (mean, SD)	0.28 (SD 0.13)	0.05 (SD 0.37)	*p* = 0.370^a^

Statistically significant *p* < 0.05 in bold

^a^Mann-Whitney U test

### Intraocular pressure

Measures of intraocular pressure were also undergone by all patients. The mean IOP was 16.07 (SD 8.81), with the “Abundant” group having the highest mean IOP (18.68, SD 2.94) (Table 2). There was no statistically significant difference in the mean IOP among groups and no correlation was found with the number of intravitreal injections (Rs = 0.021; *p* = 0.802).

## Discussion

Intravitreal injections with anti-VEGF agents are currently used to treat multiple retinal pathologies. The three most commonly used drugs are aflibercept, ranibizumab and bevacizumab. Each of these injections is administered with a different syringe and needle, which were not initially designed for intravitreal use, and contain small amounts of silicone oil. This material coats the inner surface of both syringes and needles, for smoother gliding of the plunger and facilitating injection. For this reason, the presence of silicone bubbles in the vitreous after repeated intravitreal administration of anti-VEGF drugs is studied.

The prevalence of silicone bubbles after intravitreal injections of anti-VEGF varies in the different studies. In a recent article, Melo et al. found silicone oil droplets in 25 out of 37 eyes (67.6%), although this prevalence rose to 75.7% when B-scan ultrasonography was performed [6]. Sanabria et al. reported that 89.4% of 142 treated eyes presented silicone droplets in the vitreous [3]. In our study we found that 71.7% of the treated eyes had droplets, however, in comparison to the study by Sanabria et al., our patients had a lower mean follow up time (34.7 vs. 44.7 months) and had received a lower average of intravitreal injections (10.1 vs. 16.7). Furthermore, we included patients that had only received one intravitreal injection.

These variations in the prevalence of droplets can be attributed to many other factors, such as the form of presentation, the repackaging, transport and manipulation of the drug, or even the examination technique. Recently, and by means of fluorescence labelling and imaging flow cytometry, micro-sized droplets (0.1–10 μm size range) have been detected in the vitreous of patients [5]. These cannot be detected by slit-lamp observation and recent literature point to its potential inflammatory power [6]. Another limitation of our examination technique is the different ability of quantifying for bubbles depending on media transparency, for example in the case of a cortico-nuclear cataract or a posterior capsule opacity in intraocular lenses carriers, which has not been regarded in our inclusion/exclusion criteria. Finally, the own observer ability to detect the droplets, the different use of the superfield lenses and slit lamp among physicians also can influence the results of examination.

The injection technique can also determine the amount of silicone bubbles injected. It has been shown that when the plunger is depressed to the end of the syringe, more oil penetrates the vitreous [7]. Therefore, injections that have less drug volume, such as aflibercept, transmit more silicone bubbles since the plunger has to be pushed all the way, unlike ranibizumab, in which there is a surplus of volume that stays in the syringe. The same is seen when injections are shaken to remove air bubbles prior to injection [8].

In turn, a significant correlation has been demonstrated in the literature between the number of injections and the presence of silicone oil bubbles in the vitreous [3, 4]. In our study we determined that those patients with droplets had received significantly more intravitreal injections, and that the number of injections were correlated with the number of bubbles. These findings are in agreement with Thompson et al. However, they found a weak correlation, suggesting that the amount of silicone oil in each injection could vary between the batches of syringes [4].

Bevacizumab injections with a BD 0.3 mL polypropylene syringe produce the most bubbles in our sample. Second are the injections of aflibercept with a 0.1 mL polycarbonate BD syringe, and third are the ranibizumab injections with a 0.1 mL polypropylene BD syringe. It appears that these bubbles are not detected after injections of prefilled ranibizumab [4]. In our study, despite not analyzing the prevalence of droplets regarding the agent, we could determine a moderate positive correlation between the number of aflibercept injections and the quantity of droplets, and a weak negative correlation between prefilled ranibizumab injections and the quantity of them. In 2020 Olea et al. conducted a study with silicone oil-free syringes and demonstrated absence of oil droplets larger than 25 μm [9]. Other authors, like Mello et al. have found trace amounts of silicone oil released from purportedly silicone oil-free syringes [5].

With regard to the symptoms, most patients experience floaters immediately after the injection, however their persistence is less usual. Except when the number of bubbles is greater, when these are large or aggregate between them, they cause no symptoms [3, 10]. Sanabria et al. reported that 36.7% of the patients complained of permanent floaters after injections [3]. This prevalence is in accordance with our study (39.5% of eyes). It is interesting that the prevalence of symptoms is much lower than the prevalence of droplets. Furthermore, when analyzing the disturbance of floaters in the daily life of the patients, it did not reach a score of 4 out of 10 in any of the groups. Surprisingly, the group with “Moderate” droplets in vitreous complained significantly more of floaters (60% of eyes). We believe that a reasonable cause could be that this group had a much better visual acuity than the rest, and they had also received a higher number of intravitreal injections. Thompson et al. also observed that patients with very poor VA (20/400 or less) did not complain of floaters [4].

The higher prevalence of posterior vitreous detachment in these patients must also be considered, which can be a confounding factor when assessing these symptoms. Thus, not all floaters are explained by the presence of droplets. In our study, floaters were reported in 39.5% of the patients that had droplets, however, 27.9% of the patients without droplets also complained of this symptom. Geck et al. demonstrated that 24% of the patients treated with intravitreal injections develop PVD during the follow-up [11]. In our study, 65.8% of the patients had PVD, being 88.2% in the “Abundant” group. Interestingly, a positive correlation was demonstrated between PVD and the number of bubbles. We believe that PVD allows the silicone droplets to move freely in the vitreous, thus facilitating the examiner to visualize them.

Other complications described in association with the presence of these droplets are ocular inflammation and ocular hypertension [12]. In previous studies in retinal surgeries, it has been shown that if silicone oil is kept in the vitreous for more than 12 to 18 months, it can be trapped by the cells of the trabecular meshwork, as well as by the Müller cells of the retina. For this reason, it has been hypothesized that direct obstruction of the trabecular meshwork due to the presence of high molecular weight proteins and the passage of these silicone bubbles into the anterior chamber could be a cause of increased intraocular pressure [9, 13]. Our study analyzes for the first time differences in IOP in relation to the quantity of droplets seen in vitreous. Although we did not observe statistically significant differences, the group “Abundant” had a higher mean IOP than the rest of the groups. A further study, with a longer follow-up time, anterior chamber inspection, gonioscopy and data of IOP values prior IVI treatment could be of interest to confirm this trend and to determine the presence of silicone oil droplets in the trabecular meshwork of these patients.

## Conclusions

We found a high prevalence of silicone oil droplets in the vitreous of our patients in the slit-lamp examination. However, the majority of patients did not report any symptomatology, especially those with poor VA. Moreover, we did not see a significant relation between the IOP and the number of bubbles. Further studies are needed to examine the prevalence of micro-droplets and eventually assess the safety of syringes containing particles of silicon oil.

## Data Availability

All data relevant to this manuscript is included in the present paper, but further details and raw data can be provided up to reasonable request by contacting the corresponding author.

## Abbreviations

Anti-VEGF: Anti-Vascular Endothelial Growth Factor
IVI: Intravitreal injections
IOP: Intraocular pressure
PVD: Posterior vitreous detachment
AMD: Age-related macular degeneration
ANOVA: Analysis of variance
SD: Standard deviation
DME: Diabetic macular edema
RVO: Retinal vein occlusion
VA: Visual acuity
